# The Rôle of the Central Nervous System in Disease: Recent Experimental Work in the U.S.S.R.
*A paper read to the Bath and Bristol Branch of the British Medical Association on Wednesday, 31st March, 1937.


**Published:** 1937

**Authors:** F. Bodman


### THE RÔLE OF THE CENTRAL NERVOUS SYSTEM IN DISEASE: RECENT EXPERIMENTAL WORK IN THE U.S.S.R.*

BY

F. Bodman, M.D.

If the reader should ever visit the U.S.S.R. one of the many amenities he may miss will be those civilized animals the cat and dog. Miss Delafield in her amusing book about the Soviet, *Straw without Bricks*, has suggested that this is due to the shortage of food in the years preceding Lenin's New Economic Policy, but my private theory is that all these friends of man found their way to Pawlow's laboratory. There is no doubt, however, that there was a serious dearth of experimental animals in Leningrad about this time, as serious as the shortage of anatomical subjects that provoked the exploits of Burke and Hare at the beginning of the last century.

Thus in the winter of 1923 a slight dark man, whose name was Speransky, sat in Pawlow's laboratory deploring the fact that so many of the dogs which had undergone local decortication in preparation for experiments on conditioned reflexes were dying of epilepsy: he attributed this to the adherent scars following the operation, and devised a new method of putting the cortex out of action by using a carbon dioxide snow applicator.

* A paper read to the Bath and Bristol Branch of the British Medical Association on Wednesday, 31st March, 1937.

This technique was found to be even more lethal than the surgeon's scalpel, but some very interesting results emerged. I pass by the interesting study of epilepsy and ether convulsions, which is outside our subject to-night, and draw your attention to the after-history of these animals, whose fate was not uniform.

The first and largest group of dogs died with symptoms of epilepsy during the actual procedure. The animals in the second group recovered rapidly and completely. The third group is made up of dogs which made an apparent recovery lasting from a week to several months and then died in an epileptic fit. Finally, there is a small fourth group, the dogs in which pass out of the convulsive stage into a new form of illness. As the fits disappeared tonic cramps took their place, and later a spastic gait. The dog became thinner, listless, and refused its food : paralysis of the hind legs set in, followed by loss of hair, eczema, sores and ulcers, and finally the animal died. Post-mortem, an encephalitis was discovered, with marked perivascular cuffing, resembling that seen in encephalitis lethargica.

Speransky came to the conclusion that the irritating agent was conveyed not by the blood but by the cerebrospinal fluid, and embarked on a series of experiments which demonstrated the solvent action of the cerebrospinal fluid on isolated brain substance. The products of disintegrating brain substance autolysed in cerebrospinal fluid proved "toxic" when injected intrathecally into normal dogs. The resulting symptoms resembled in some cases those of hydrophobia, in others those of tetanus. The same results were observed on injecting cerebrospinal fluid from an animal whose cortex had been frozen. This resemblance in symptomology led Speransky to investigate the rôle of the cerebrospinal fluid in the spread of rabies virus and tetanus toxin through the brain. A collaborator, Sheshkov, devised an ingenious operation whereby inoculation with rabies or tetanus toxin was carried out into a walled-off portion of the subarachnoid space. The result was a great lengthening of the incubation period, and Speransky came to the conclusion that the circulation of the cerebrospinal fluid played an important part in the spread of neurotoxins. This, of course, is contrary to the belief currently held that rabies virus invades the central nervous system solely by the nerve trunks.

The next step was to work out the circulation of the cerebrospinal fluid. At that time the Russians were ignorant of the recent advances in knowledge of its physiology, and though Speransky refers to the work of an American, Weed, on this subject, that of Dandy had not attracted his attention. Unfortunately, national barriers had prevented that diffusion of knowledge which is so desirable, and valuable time and energy were wasted in reduplicating research—perhaps not altogether wasted, as results were confirmed and some new facts added, notably the rôle of the nasal lymphatics in the absorption of cerebrospinal fluid.

Having worked out the method by which toxins and irritants were conveyed by the cerebrospinal fluid, the Russians turned their attention to the transfer of irritants along nervous tissue itself, and demonstrated that both neurotropic and indifferent substances could pass along nerve trunks, that penetration into the nerve trunk takes place most easily at the nerve endings, and that movement along the nerve trunk depends on muscular contraction.

In the course of the experiments on the movement of irritants along nerve trunks in some cases irritants were injected into nerve trunks, in others the nerve trunk was severed and the irritant applied to the cut surface. In these latter cases " trophic " disturbances developed.

After a few preliminary trials a technique was devised by Vishnevsky which produced trophic ulcers in every case. Under narcosis the sciatic nerve in the middle of a dog's thigh was divided. Pus was rubbed into the central divided end and the wound was sewn up. After this cerebrospinal fluid was extracted, and this extraction *repeated* every two to five days. About seven to twelve days later a fluctuating swelling, loss of hair, and finally ulceration occurred on the corresponding foot-pad. The ulcer rapidly enlarged, involving tendons, joints and bones. All this occurred in the midst of completely healthy tissues, unconnected with any inflammation.

After a further period (from five days to eight weeks) a similar non-healing ulcer appeared on the symmetrically situated site of the other (unoperated) limb. If before ulceration occurs the fluctuating swelling is incised a sanious fluid escapes. The dystrophy begins in the underlying parts. I can confirm this clinically. The other day I had in my wards a man with diabetic neuritis and a perforating ulcer of the right foot. On radiographing the left foot a decalcified area in the shaft of the second and third metatarsals was discovered, which was probably the first stage of a perforating ulcer of the other foot.

Was the trophic ulceration due to septic infection of the nerve trunk ? Fresh experiments were carried out by a team of workers who used, instead of pus, bile, mustard gas, croton oil, acids, carbolic acid, formalin, and fresh emulsions of normal organs. The same symmetrical trophic ulcers were obtained. The results were not therefore a consequence of an infectious myelitis.

A further demonstration that the nervous system was disorganized as a result of injury to one nerve trunk was planned. Two animals were chosen, of which one served as a control. In both the right and left sciatic nerves were exposed under narcosis and divided. In the experimental animal the left nerve was resutured, into the central end of the right nerve croton oil was injected. In the control animal the left nerve was resutured, but no irritant was applied to the divided end of the right nerve. This experiment was carried out on several pairs of animals, who were subsequently killed and the sutured left sciatic nerves were examined under the microscope. In each case the experimental animal whose contralateral nerve had been irritated showed disorderly matting of new fibres. Only a few fibres crossed the line of suture. The controls showed vigorous regeneration of the axis cylinders and ultimately a completely restored nerve. The irritant at a remote spot had effectively arrested the repair of a nerve in the contralateral segment. Under these conditions trauma of a nerve appears to set up an unusual form of nerve irritation, which may not only distort the function of the whole nervous system but may even destroy it.

You will remember that the results of freezing the cortex differed considerably: some animals died, some recovered, some relapsed into a chronic illness. Here also, under exactly regulated conditions, the consequences differed in degree and time of development. But in these experiments there was always a latent period during which the animal appeared normal. Yet within its nervous system a hidden process was developing. What, says Speransky, is the difference between this latent period and the "incubation" characteristic of infections, and supposed to be due to the first stage of the struggle between the germs and the host?

He records a further experiment, which reminds us of orchitis appearing as a complication of mumps. In rabbits the sciatic nerve was exposed at the level of the origin of the pudendal nerve. This nerve was touched with a solution of lewisite. The wound healed by first intention. After a latent period of three days a blue spot appeared on the homolateral scrotum. The spot increased in size and the testis swelled. A few days later the tissues became normal again, but now the contralateral scrotum and testis began to swell.

Here we have, after irritation of a nerve, a general latent period, during which changes in the nervous network cannot be detected. After local manifestations have appeared this process continues, and periodically throws out at the periphery, proof of its development.

It cannot be maintained that the perforating ulcer is the real illness. But the trauma to the nerve brought about not only a peripheral irritation but some pathological change in the central nervous system.

If excision of the peripheral ulcer is performed this will not arrest the progress of the nerve dystrophy: new ulcers will form eventually. This work also explains the frequency of relapse of perforating ulcers after sympathectomy. A new trauma to the nervous system will aggravate the process.

If, then, the appearance of the perforating ulcer is an end result, what has been taking place in the nervous system during the latent period ? It is after all only a latent period because of the imperfection of our indicators. Fresh experiments were carried out by a team of nine workers whose names I will not attempt to reproduce, and a histological study made of the nervous system of these animals. Some interesting data emerged from these studies :—

First, that changes can be discovered not only after direct introduction of an irritant into the nerve trunk, but also after its application to the skin or after intramuscular or subcutaneous injection.

Secondly, that the changes did not always correspond to the concentration of the agent used. Weak concentrations generally produced the most marked changes. This appears to confirm the idea that the effects are due to the peculiar dystrophy set in train by the irritation rather than a direct effect of the irritant. The picture was constant whether a chemical or bacteriological insult had been employed. Changes were found in the nerve trunk, in the posterior root ganglia, in the spinal cord, and in the sympathetic chains. It was constantly observed that only some of the cells altered. It is significant that of two cells lying side by side one was so altered that it was considered dead, the other appeared absolutely normal. Such instances prove that these effects are not the results of direct action of the foreign irritant. For why, if this was so, should one cell be killed and another a few microns away remain unscathed ? This can only be understood if it is recognized that the cause of death of the cell is a distortion of the relations between this cell and other neurones that have altered their functions under the influence of irritation.

Moreover, it was found that after chemical trauma to a fore-limb changes were pronounced in the cervical and lumbar enlargements of the spinal cord, but the thoracic segments were unaffected. If the process was due to the mere spread of an irritant how do extensive tracks escape ? Did this mean that there was a special, hitherto unknown, defensive mechanism in the central nervous system ? Could a chemical " blow " to one nerve cause the whole nervous system to put itself " on guard "—to develop barriers across which harmful agents had not yet travelled ?

A new series of experiments was devised. For each test two dogs were selected. One was given a small dose of formalin into the left sciatic nerve. The other dog remained as a control. After a fortnight both dogs were inoculated with laboratory rabies into the right sciatic nerve. Of the dogs that had suffered chemical trauma more than 50 per cent. failed to develop rabies. The remaining odd 40 per cent. developed it after a long incubation period. Of the controls only one out of eleven failed to develop rabies.

These experiments uncover a process which Speransky calls a nervous dystrophy; it can be initiated by a foreign irritant, which does not kill nerve cells, but sets in train a sequence of changes which may result in the actual death of tissues at a site far removed from the initial trauma.

Further studies of experimental rabies were made. It is known that anti-rabic serum is *in vitro* a powerful serum and yet *in vivo* has very little action. It has further been established that anti-bodies of specific sera do not pass from the blood into the cerebrospinal fluid. This is due to the special selective permeability of the choroidal plexuses, the "blood-brain-barrier." In order to bring the anti-rabic serum from the blood into contact with brain tissue this barrier must be destroyed. The most effective method of destroying this barrier was worked out by Vishnevsky and has been called "pompage." If as much cerebrospinal fluid as possible is extracted from a dog (using a syringe to complete the extraction), re-injected into the theca, and then extracted again, the fluid will no longer be colourless. As the process is repeated it becomes yellow and then red. If the procedure is continued the fluid in the cerebral ventricles becomes blood-stained and hæmorrhage occurs in the cerebrum.

This method of pompage was used in a series of experiments to bring anti-rabic serum from the blood into the brain. Both experimental animals and controls were intracerebrally inoculated with rabies, and three hours later received anti-rabic serum intravenously. The experimental animals received pompage; the controls did not. All the controls died: the experimental animals remained healthy with few exceptions. These results are very significant, as this is the first time in the study of rabies that anti-rabic serum constantly protected against rabies.

These successes led to further applications. Diphtheria was chosen. Rabbits were selected in pairs. Fifteen minimum lethal doses of diphtheria toxin were given intravenously, and three quarters of an hour later 1,850 international units of anti-toxin were given intravenously: pompage was carried out on one animal in each pair after fifteen minutes. All the controls died: all the animals who underwent pompage survived. Speransky considers that these experiments prove that the cause of death in diphtheria is the poisoning of the *nervous* system, and that if the *nervous* system can be protected by anti-toxin there need be no anxiety about other organs. The same amount of anti-toxin supplied by intravenous injection to the heart and suprarenals proved useless as long as the central nervous system could not be reached by the anti-toxin.

Further experiments were made with tetanus toxin, but the experimental animals outlived their controls by only a short period. Speransky blames the poor quality of his serum.

Rabbits are very sensitive to dysentery toxin, which produces not only digestive disturbances but convulsions and paralysis. Again, anti-dysenteric serum protected only those animals treated by pompage.

As Speransky rather sadly observes, many infections cannot be reproduced in animals, and he almost apologizes for introducing some clinical researches from the wards. At the time of these researches there was no concentrated anti-scarlatinal serum available, and for adults the usual dose of the weak serum was 100—200 c.cm. In the light of the previous experiments it was considered that the need for so large a volume of serum might be that it served to bring about a "leak" in the choroidal plexuses. Would it be possible to obtain a curative effect by intrathecal injection of very small doses? Speransky asked the director of the Fever Hospital in Leningrad to make some investigations.

Instead of giving 200 c.cm. intramuscularly, from 4 to 10 c.cm. of unconcentrated serum were introduced by lumbar puncture. The serum was used only in severe cases. The mortality in the patients treated by intramuscular injections of 100—200 c.cm. was 20 per cent. The mortality in those receiving intrathecal injections of 4 to 10

c.cm. was 10 per cent.* The clinical effects of the introduction of small quantities of serum intrathecally were the abolition of delirium, the disappearance of the rash, and the decrease of the redness and swelling of the throat. The *significant* feature is that doses of serum that proved ineffective when introduced by the blood to the diseased organs themselves put an end to the pathological processes in these organs when applied to the central nervous system directly.

A further clinical experiment was made with convalescent serum in measles. A small quantity given intravenously had no protective effect. The same quantity introduced intrathecally protected against measles. What then, asks Speransky, are measles and scarlatina if blockade of the nervous system prevents or abolishes all the symptoms of these diseases ? He argues that if all the symptoms disappear, owing to reactions in the central nervous system, then logically the central nervous system does not simply participate in the disease, but is itself the organizer of the external manifestations.

Confirmation of this view was obtained in a series of experiments with diphtheria toxin. Engorgement of the suprarenal is a typical post-mortem finding in diphtheria, especially in guinea-pigs. Large quantities of anti-toxin were administered intravenously in guinea-pigs : diphtheria toxin was injected shortly afterwards, intrathecally in some, intravenously in others. The guinea-pigs receiving intravenous toxin had generally no suprarenal changes : every molecule of toxin in the blood was neutralized by the anti-toxin there. But the guinea-pigs receiving diphtheria toxin intrathecally showed extensive changes

* The author supplies no information to enable us to decide if this difference is significant.

in the suprarenals. Here the anti-toxin in the blood could not pass the barrier to neutralize the toxin in the central nervous system; and although the suprarenals were flooded with anti-toxin, yet the changes took place. In other words, the cause of the suprarenal affections in diphtheria is not the local action of the toxin, but the altered relations between the tissues and the poisoned nerve centres.

The first application of "pompage" in the wards was carried out at the Fever Hospital in Leningrad in cases of cerebrospinal fever. It is known that auto-immunization to the meningococcus commences at the same time as the disease, and after two to three weeks should be considerable; nevertheless, the disease by this time is at its climax. If the anti-bodies in the blood could be transferred to the central nervous system recovery should be accelerated. The subjects were seven children, each of whom had already had several lumbar punctures to relieve intracranial pressure. Pompage was carried out, extracting 10 c.cm. of the fluid and re-injecting it from five to eight times, the last time pouring the fluid away, and repeating the procedure from five to ten times. Five of the patients improved, the turning-point of the disease coinciding with the institution of pompage; the sixth recovered, but the improvement could not be connected with pompage; the seventh died, possibly as a result of the pompage. There was no doubt that in some cases pompage had a favourable effect. But doubt arose as to whether its value depended on the mobilization of anti-bodies through the blood-brain-barrier, or whether the actual trauma to the nervous system caused by the pompage did not set in train a biological reaction in the nervous system.

Obviously the next task of the Russians was to map out the range of symptoms brought about by the dystrophy, and to investigate what relations obtained between trauma to individual nerves and the resulting dystrophy. For these experiments rabbits were used. Remembering the keratitis that is known to follow section of the trigeminal nerve (for tic douloureux), a start was made by injecting croton oil into the infraorbital branch of the fifth nerve, after which the nerve was divided in the middle of the injection site. Within a few days bilateral conjunctivitis developed, and on the operated side keratitis and corneal ulceration frequently followed. An indolent ulcer appeared on the lip in one or two days, followed by an ulcer on the corresponding half of the other lip, and later symmetrical ulcers of the tongue. All these conditions gradually subsided, but in a proportion of cases, after one to two months, a condition resembling dental caries was noticed, first on the operated side, later on the sound side. Two rabbits died of otitis media.

On repeating the experiment with dogs the same changes were observed, and in addition herpetic eruptions around the mouth and hæmorrhages in the lung, and in the mucous membrane of various parts of the digestive tract. These hæmorrhages reminded the investigators of the hæmorrhages reported to have occurred after destruction of the hypothalamus. Were the hypothalamus and infundibulum involved in this dystrophy ? What would happen if this part of the brain were irritated ?

This question provided a technical problem of some magnitude even to these operators, skilled in the cerebral surgery of animals. But the difficulties were successfully overcome, and a small glass sphere or ring, of the size of a pea, was inserted behind the sella turcica without injury to the rest of the brain. In this position the foreign body presses on the tuber cinerium, mammillary bodies and posterior perforated spot, without pressing on the pituitary. This operation was carried out on about 100 animals. The duration of survival varied within wide limits, from ten hours to a year or more. In spite of these differences the post-operative symptoms were remarkably constant.

Within a few hours hæmorrhages appeared in the gums, resembling those of scurvy, and later ulcers of the lips, tongue and hard palate. On two occasions ulcers appeared in the cheek, exactly resembling the cancrum oris seen after severe measles or scarlet fever. Next papillomata appeared on the mucous membrane of the cheek and tongue, and later dental caries was observed. Within a few days, or sometimes six weeks later, corneal ulceration, keratitis and conjunctivitis occurred, and on several occasions purulent rhinitis and otitis media. Another extraordinary phenomenon was recorded: ordinarily the stitches were removed on the eight day, but sometimes, without any preceding inflammation, the operation wound, on removal of the sutures, gaped open widely. Another skin change was the loss of hair. In the internal organs hæmorrhages were frequently found in the lungs, sometimes so dense that they gave the impression of red hepatization. Constant and characteristic changes were found in the intestinal tract. These were hæmorrhages and ulcerations, of remarkably constant situation. In the stomach they are found in the pylorus and in the duodenum. The changes are sparse in the small intestine, are again found at the ileo-cæcal valve, but are absent until the rectum is reached, where the changes are again observed.

Even a hasty review of the changes described shows that the lesions following the glass sphere operation on the tuber cinereum correspond nearly exactly to the changes following irritation of the fifth nerve. It was doubtful, therefore, if there was a *direct* connection between the nerve trauma and the resulting peripheral changes. Was some intermediate process in the nervous system initiated by the damage to the nerve ? If that was the case, trauma to nerves at remote points should produce identical peripheral disturbances. This was fully confirmed by subsequent researches.

Chemical traumata to the ganglia of the sympathetic chain, insertion of irritants into the pulp cavity of teeth, traumata of the spinal cord or sciatic nerve, were followed by changes in the oral and nasal cavities and in the usual sites in the gastro-intestinal tract. Speransky considers that these experiments confirm the thesis that any *portion of the nervous system can become the starting-point for processes of a neurotrophic character.* The nervous system is a complete unit in which local interference affects the whole. The changes produced pass away gradually, but not completely, and new adaptions are made resulting in a new equilibrium. After the local lesion the nervous system is a different unit, and reacts to stimuli in a new manner.

Not all the animals subjected to the glass sphere operation developed the complete picture of generalized dystrophy ; and after a large number of observations the material could be divided into groups, comparable to those described at the beginning of this paper, following freezing of the cerebral cortex :—

1. Fulminating development of dystrophy—death in a few hours.

2. Acute illness—death in a few days.

3. Acute illness, followed by apparent recovery.

4. Acute illness, followed by recovery and then a series of relapses, ending in death.

5. No illness followed the nerve trauma, but on subjecting these individuals to a further trauma the symptoms expected from the first trauma rapidly developed.

6. No illness followed the first "blow," and repeated traumata failed to initiate a dystrophy.

These variations must be due to the individual properties of each animal. Already Pawlow had found in his work on conditioned reflexes that even after most accurate standardization of environment results were so variable that it was necessary to propound a theory of types of nervous constitution in dogs.

Classes 4 and 5 demonstrate that disappearance of symptoms or absence of symptoms after the first blow is no proof that the nerve elements have recovered. A new pathological focus has been created in the nervous system, which alters the relations of the whole. To foresee possible deviations from normal reactions, it is necessary to know the history of each individual nervous system. Speransky quotes the aphorism: "There are no illnesses, there are only ill people." Rather a paradoxical tribute to individualism from a Communist state !

If the origin not only of the primary changes but also of the relapses was to be found in agencies of a nervous character, then there must have been established in the nervous system of the organism some pattern which continued to retain traces of the past injury. Could this be the underlying explanation of "sensitization" and "dormant infection"? A

series of investigations of such chronic diseases as tuberculosis and syphilis was planned, and without going into details I can summarize them by saying that the spread of tuberculosis in experimental animals depended on the site of injection, and could be intensified or inhibited by simultaneous irritation of nervous tissue, or by denervation of organs in which the infection was expected.

I must refer in detail to one very interesting research which demonstrates beautifully the thesis Speransky was attempting to confirm. It has long been a matter of dispute as to the route by which infection spreads from or to the kidney, testis and epididymis. Various theories have been put forward, incriminating the blood, lymph, urine and semen. Now Speransky, arguing from previous experience, believed that the formation of new lesions was not accidental, but pre-determined by tissue derangements already provoked by nervous influences. Was it possible to bring about a secondary focus in an organ without interfering with it or its nervous supply?

He selected the kidney in rabbits as one of the most difficult sites. Usually rabbits succumb to generalized tuberculosis before the kidneys are affected, and only rabbits surviving over three months show tuberculous renal lesions. In his experiment tuberculous lesions of both kidneys were found four to five weeks after injection of tubercle bacilli into the testis. His explanation is that by irritating the nervous system of the testis the closely-related nervous system of the kidney was involved, and the resulting derangement prepared the ground for the subsequent infection by tubercle bacilli.

To demonstrate that this was not a selective action of the tubercle bacillus the experiments were repeated by another investigator. This time staphylococci were injected into the ovary. The results of unilateral infection of the ovary were bilateral staphylococcal abscesses of the kidneys. These experiments were confirmed again by two further members of Speransky's team, who then introduced a further refinement. They injured the ovary, not with bacilli but with two drops of 2 per cent. formalin. Three weeks later they gave an intravenous injection of staphylococci, and a month later the animals were killed. Post-mortem they found a few adhesions round the ovary; no changes were found in any other organs except the kidneys, where multiple abscesses were always discovered. The localization of lesions depends not on any selectivity of the bacteria but on processes of a nervous nature. In all these experimental infections not only is a depôt of live microbes established but a trauma is inflicted, which initiates the nervous dystrophy. The two processes, infection and dystrophy, proceed simultaneously, the trophic changes in remote tissues preparing the site for further infective foci. We must conclude from the experiments that up till now we have over-estimated the importance of bacteria, and under-estimated the capacity of the host to inflict damage on his own tissues.

This co-operation between infection and nervous dystrophy has been further illustrated by the study of experimental syphilis in rabbits. Normally 95 per cent. of rabbits when inoculated in the scrotum develop a chancre-sclerosis in five weeks. This sclerosis progresses up to a certain point, and then disappears. After a pause secondary manifestations appear in the eyelids, nose, lips and anal region.

The next experiment was to infect with syphilis a scrotum partially denervated by section of the pudendal or genito-femoral nerve. Two results were apparent. The incubation period of the chancre, instead of the usual five weeks, was prolonged to from seven to ten weeks (in one case thirteen weeks), and the sclerosis was much less marked. But here comes the interesting finding: in half the cases, after a latent period, a symmetrical chancre appeared on the uninfected scrotum. This appeared in from ten to twenty weeks after the original infection, and this symmetrical chancre contained spirochætes. These experiments are analogous to the symmetrical trophic ulcers of the feet obtained by traumatizing the sciatic nerve.

These observations of Speransky seem to me very suggestive. The controversies about the causal organism in rheumatic fever and influenza resolve themselves easily if his thesis is accepted. Schlesinger's virus insults the nervous system of the rheumatic child, and this is followed by foci in the tonsils, which are favourable for the streptococcus. A further latent interval and lesions, Aschoff nodules (apparently sterile), manifest themselves in the heart and joints. Again, in influenza, there seems to be no doubt that the virus of Andrews, Laidlaw and Smith is the initiation of this illness. But it must have been your experience, as it was mine in this last epidemic, that the few cases of bronchopneumonia produced an almost pure culture of bacillus influenzæ in the sputum.

An infection, exactly like a chemical irritant, may produce both local and distant changes in the tissues. These may resolve without trace ; but if the nervous system is involved a new process commences, and the original irritant becomes obscured and loses its importance.

Speransky states that since the time of Virchow and the exponents of cellular pathology the rôle of the nervous system in inflammation has been denied. He maintains that the nervous system is the fundamental organizer of the inflammatory process.

I have not time to recount to you in detail his researches in acute inflammation. But he maintains that the old controversy concerning the rôle of the nervous system in inflammation must be decided in favour of the nervous system playing a controlling part.

The time has come to review the situation brought about by these researches, which have demonstrated that these neuro-dystrophic processes may to a degree depend on the site of the initial trauma, and to some extent on the character of the inflammation. Immunological reactions cannot alter the development of these processes. However efficient a serum is, it cannot do more than eliminate the exciting agent. It is doubtful, for example, whether recoveries from cerebrospinal meningitis depend on the immunological reactions.

The discovery of the microbe by Pasteur was a great advance, but it has artificially simplified reality. Pathological processes have been attributed to the microbe, which were really due to the reaction of the nervous system of the host.

The microbe has three possible rôles. It may initiate a process, as in anthrax, plague or glanders. It may act as a catalysing agent, to facilitate a process already prepared beforehand by other agents (as the streptococcus in rheumatism.) Or it may appear merely as an indicator in tissues already altered in a direction favourable for this microbe and no other (spirochætes in cancrum oris). The microbe may change its rôle and start as an initiator and end as an indicator. This is seen in syphilis. To cure syphilis the neuro-dystrophic process must be arrested; then the spirochæte will automatically disappear. Antibody-antigen reactions are as physiological as the secretion of saliva when food is put in the mouth. As Speransky says, disease is not the struggle between the host and the parasite.

Two parallel processes are developing side by side. One, the antigen-antibody reaction, which is as normal a physiological function as salivation; the other a process whose manifestations transcend the bounds of normal physiology. Incubation is observed in both processes, but it has a distinct time relation in each. The experiments related above show that incubation is not a special property of micro-organisms or even foreign proteins. It occurs in connection with simple chemicals, since this phenomenon depends not on the irritant but the irritated organism. The experiments suggest that incubation can be defined as the time taken for the irritated nervous system to establish a new equilibrium.

Our present therapeutic objective is the abolishing of pathological phenomena. We act as if we imagined disease as something foreign which must and can be expelled: "The devils must be exorcised." This causal approach, considering the process as the collision and subsequent interaction of two separate factors, is fallacious. What happens is not a disease in the organism, but the emergence of a new organism.

This is not the only concept we must abandon if we are to accept Speransky's interpretation of his researches. I have had no time to refer to a series of researches on tetanus, which raise acute doubts on the question of the quality of excitation in nerve tissue. The sanctified view is that the state of excitation conducted along the nerve is always the same. But it is possible that the "all or nothing" principle and the principle of the uniformity of nerve impulses may have to be liquidated.

Sir Thomas Lewis in his recent lectures on the nocifensor system of nerves has had to postulate a hitherto unsuspected system of nerves to account for a series of reactions, including hyperalgesia and flare: he denies the existence of trophic nerves. The Russians started out believing that they would discover a special system of trophic nerves, but as their researches extended they found that there was not a pathological process into which the nervous system did not enter. Acute and chronic inflammation, new growths and ulcerations, all were organized by the nervous system. There is no separate trophic nervous system; the maintenance of the eutrophy of the tissues is a function of every part of the nervous system. As Sherrington has said, all nerves are trophic nerves.

The cellular pathology of Virchow, the bacteriology initiated by Pasteur, the chemotherapy of Ehrlich, have each contributed to progress. But none of these ideas provides a complete answer to the problems of disease. The study of the nervous component of pathological processes gives a conception of the main link in the sequence of events, and establishes the principle of integration of all the manifold pathological forms into a single system.

I must leave my thesis incomplete, but I hope that I have done justice to Speransky in this very brief report of researches which may prove fundamentally important. Three years ago in Moscow I met Speransky. From my conversation with Russian doctors I had already realized that to them he was a hero, admired in the same way that Lister was admired by our grandfathers and Pasteur by the French. I was impressed by his modesty and friendliness, by his energy and capacity to inspire his colleagues, and I look forward to the day when political barriers will no longer prevent him from travelling to England and telling us himself of his work in unravelling the problems of disease.

### BIBLIOGRAPHY.

A. D. Speransky, *A Basis for the Theory of Medicine*, Moscow, 1935.
Weed, *Amer. J. Anat.*, 1923, vol. xxxi., p 191.
Dandy, *Ann. Surgery*, 1919, vol. lxx., p. 29.
Vishnevsky, *Zeitsch f. d. ges. exp. Med.*, 1928, vol. 63, Nos. 5, 6.
Lewis, *Brit. Med. Journ.*, 1937, vol. i., 493.
Sherrington, *Schäfer's Text-book of Physiology*, 1900, vol. ii., p. 876.

